# A 12-Week Vigorous Exercise Protocol in a Healthy Group of Persons over 65: Study of Physical Function by means of the Senior Fitness Test

**DOI:** 10.1155/2016/7639842

**Published:** 2016-05-03

**Authors:** Francesco Todde, Franco Melis, Roberto Mura, Massimiliano Pau, Francesco Fois, Sara Magnani, Gianfranco Ibba, Antonio Crisafulli, Filippo Tocco

**Affiliations:** ^1^Department of Medical Sciences, Sport Physiology Laboratory, University of Cagliari, 09124 Cagliari, Italy; ^2^Department of Mechanical, Chemical and Materials Engineering, University of Cagliari, 09124 Cagliari, Italy; ^3^Italian National Olympic Committee (CONI), Sardinia, Italy

## Abstract

The aim of this study was to assess the effects of vigorous exercise on functional abilities by means of a Senior Fitness Test (SFT) in a group of elderly adults. Twenty healthy and inactive people performed vigorous exercise (VE: 12 men and 8 women, aged 69.6 ± 3.9 years). At the beginning of the study (T0) and after 3 months (T1), each subject's functional ability was tested for muscular strength, agility, cardiovascular fitness, flexibility, and balance. The VE was designed with continuous and interval exercise involving large muscle activities. Functional exercises were performed between 60% and 84% of heart rate reserve (HRR) for a duration of 65 minutes. Five out of the 6 SFTs performed were found significantly improved: Chair Stand (T0 12.4 ± 2.4, T1 13.5 ± 2.6, *p* < 0.01), Arm Curl (T0 14.2 ± 3.6, T1 16.6 ± 3.6, *p* < 0.01), 2 min step (T0 98.2 ± 15.7, T1 108.9 ± 16.2, *p* < 0.01), Chair Sit-and-Reach (T0 −9.9 ± 7.7 cm, T1 1.7 ± 6.3 cm, *p* < 0.01), and Back Scratch (T0 −15.8 ± 10.9 cm, T1 −8.4 ± 13.1 cm, *p* < 0.01). Our results suggest that a high intensity protocol and functional exercises can improve functional mobility and muscle endurance in those over 65 years of age. SFTs are an effective method for assessing improvements in the functional capacity of elderly adults.

## 1. Introduction

It is well known that exercise in the older population may prevent several diseases [1–4]. Reduced physical activity impairs the quality of life in elderly people with Alzheimer's Disease [4], Parkinson's Disease [5], and Depressive Disorders [6]. Moreover, musculoskeletal, cardiopulmonary, and cerebrovascular decline are associated with poor physical fitness because of the cumulative effects of illness, multiple drug intake, fatigue, and bed rest [7, 8]. The effects of physical activity and exercise programs on fitness and health-related quality of life (HRQOL) in elderly adults have been widely studied by several authors [9–11]. De Vries et al. [11] conducted a meta-analysis focusing on elderly patients with mobility problems and/or multimorbidity. Eighteen articles describing a wide variety of actions were analyzed. Most used a multicomponent training program focusing on the combination of strength, balance, and endurance training. In 9 of the 18 studies included, interventions were supervised by a physical therapist. Intensity of the intervention was not reported and the duration of the intervention varied from 5 weeks to 18 months. This meta-analysis concluded that, considering quality of life, the exercise versus no-exercise studies found no significant effects. High-intensity exercise appears to be somewhat more effective in improving physical functioning than low-intensity exercise. These positive effects are of great value in the patient population but the most effective type of intervention remains unclear. Brovold et al. [7] recently examined the effects of high-intensity training versus home-based exercise programs using the Norwegian Ullevaal Model [12] on a group of over-65-year-olds after discharge from hospital. These authors based their study on the Swedish Friskis-Svettis model [13] which was designed by Johan Holmsater for patients with coronaropathy to promote their return to work and everyday activities and improve their prognoses. This model includes three intervals of high intensity and two intervals of moderate intensity, each one lasting for 5 to 10 minutes. Included in each is coordination. Exercises consist of simple aerobic dance movements and involve the use of both upper and lower extremities to challenge postural control [13]. Exercise intensity was adjusted using the Borg Rating of Perceived Exertion (RPE) Scale. Moderate intensity was set between 11 and 13, and high intensity was set between 15 and 17 on the Borg Scale.

Thus, little is known about the effects of monitored vigorous exercise in elderly people. While significant benefits for basic motor tasks (such as balance and gait) can be achieved through different kinds of physical activity (i.e., stretching exercises, treadmill, Pilates, and strength and balance training), no conclusive relationship has been proven between its intensity and such improvements. Recently, Pau et al. [14] reported that spatiotemporal gait parameters and sit-to-stand performance significantly improve through vigorous (but not light) exercises, thus suggesting that higher levels of intensity might be more suitable in generally improving static and dynamic daily motor tasks.

On the basis of the aforementioned consideration, this study aimed to evaluate the effect of monitored vigorous exercise (VE) on several functional capacities by applying the Senior Fitness Test (SFT) [3] in a group of healthy over-65-year-olds.

## 2. Methods

### 2.1. Experimental Approach to the Problem

Recruitment criteria were one or both of sedentariness and dysmetabolism. Thus, we selected subjects who were not physically active or involved in any exercise program; that is, they had a sedentary lifestyle. Moreover, before entering the study, they were carefully screened for metabolic problems which attested a dysmetabolic status, as increased levels of plasma glucose, free fatty acids, triglyceride, and urate in fasting state. Both criteria were verified by means of family doctor databases of subjects.

Exclusion criteria included major diseases or conditions such as severe heart disease, uncontrolled hypertension, obesity, osteoarticular pathology, and neurological disease. Criteria were evaluated on the basis of clinical history, resting ECG, and physical examination. Participants maintained their lifestyles and were instructed not to take part in any other physical programs throughout the study. At the time of the initial design, the study consisted of a 12-week randomized controlled trial with a frequency of 3 times a week, 36 sessions in all, ending with a new assessment of their wellness and the potential persistence of the results on functional/physical capacities.

### 2.2. Subjects

**Figure 1:**
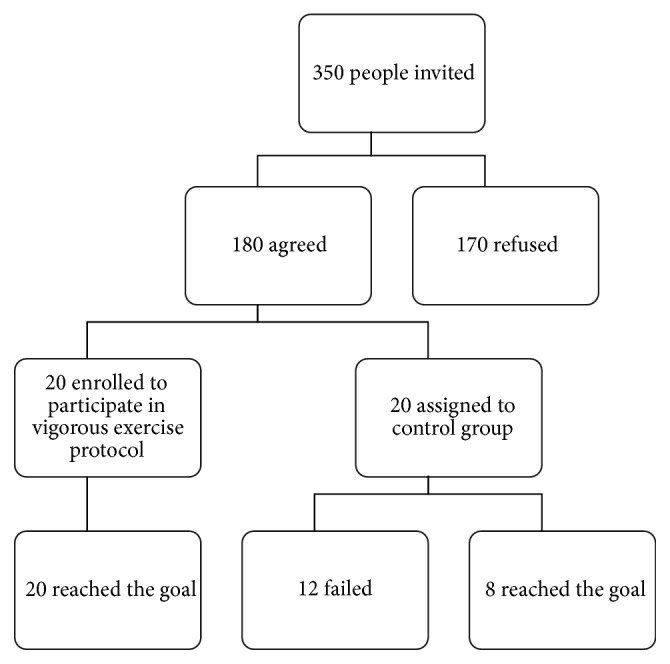
Flow chart showing the study trend.

Recruiting lasted 6 months starting from September 2013. Participants were recruited by means of family doctors to whom the goal of the study was explained. The recruitment flow chart is shown in Figure 1. Three hundred and fifty people aged ≥ 65 were invited to participate. Of these, 51.4% agreed to be included in the screening list while 48.6% refused to participate, mainly for family reasons such as illness/hospitalization/old age of a family member. Forty people were found eligible to participate in the research protocol. Randomly, twenty were assigned to VE and twenty to the control group. The latter were instructed not to take part in any physical activity throughout the study period. All the selected participants signed an informed consent. The study was performed according to the Declaration of Helsinki and approved by the local ethics committee on September 23, 2013.

### 2.3. Procedures

According to the ACSM guidelines, the physical activity (PA) was set between 60% and 84% of the heart rate reserve (HRR) [9] and continuously monitored with a heart rate monitor (Polar® T31 Coded*™*) connected to a telemetry system (Hosand®).

Each participant's individual HRR was calculated according to the Estimated Maximal Heart Rate Formula [15]: HR_max_ = 206.9 − (0.67 × Age). The baseline HR values were collected from all subjects for three consecutive days in the morning right after waking up and the mean value was calculated.

Since specific characteristics of music such as rhythm and melody have been shown to provide a strong incitement to performing physical exercise [16], a Remix Pop Music compilation of the 1970s was used as a background soundtrack for the present training protocol.

### 2.4. Vigorous Exercise

The training protocol consisted of three phases:

1. *Warm-Up Phase,* up to 10 min.
2. *Active Phase*, 45 min, including mixed exercises.
3. *Recovery Phase*, up to 10 min.

*(1) Warm-Up Phase (over 60% HRR)*. Slow, dynamic movements of the lower-, mid-, and upper-body main muscle groups through the full range of motion followed by static and dynamic stretching exercises.

*(2) Active Phase (between 60% and 84% HRR)*. Continuous dynamic and interval training mode exercise involving large muscle activities with an increasing level of difficulty and intensity. Subjects began with a short walk, alternated with various step exercises (e.g., both side and forward-backward step up and down on the platform, with alternate footsteps). Then, they went on performing alternate upper-limb lifts (while keeping inferior limbs flexed) and lower limb flexions and extensions (knee lifts, both side and forward-backward leg lifts, and leg curls), as a sort of brief and easy sequence to be repeated for a fixed time. Integrated multiple plane exercises for upper and lower limbs using elastic resistances (Xertube®) completed the last part of the Active Phase. To reach the goal of gradually augmenting the intensity of the program, the coach continuously checked the HRR level of subjects who were progressively increasing the duration and the number of exercises. The resistance of the elastic bands was also increased by one level (from very light to medium) every 4 weeks.

*(3) Recovery Phase (<60% HRR)*. Postural control and spine mobility exercises in a quadrupedal position with the platform support, exercises of static balance over either 4 or 2 supports, eyes either open or closed, and with core muscle activation. The latter phase also included various poststretch exercises to restore the preexercise muscle length.

The VE was conducted under continuous HR monitoring by a professional fitness coach with a degree in Motor and Sport Science.

### 2.5. Description of the Senior Fitness Test (SFT)

In accordance with Rikli and Jones [3], we used the following tests at the beginning (T0) of the study and after 12 weeks (T1).

#### 2.5.1. 30-Second Chair Stand Test

*Purpose*. Its purpose is to assess lower-body strength needed for numerous tasks such as climbing stairs, walking, and getting out of a chair, bathtub, or car (increased ability in performing this exercise may reduce the possibility of falling).

*Description*. It consists of a number of full stands from a seated position which can be completed in 30 seconds with arms folded across the chest.

#### 2.5.2. 30-Second Arm Curl Test

*Purpose*. Its purpose is to assess upper-body strength needed for performing household and other activities involving lifting and carrying things such as groceries, suitcases, and grandchildren.

*Description*. It consists of a number of biceps curls that can be completed in 30 seconds, holding a hand weight of 5 pounds (2.3 kg) for women and 8 pounds (3.6 kg) for men.

#### 2.5.3. 2-Minute Step Test

*Purpose*. It is an alternative aerobic endurance test to be used when time restraints and/or space limitations impede administering the 6-minute walk test.

*Description*. It consists of a number of full steps completed in 2 minutes, raising each knee to a point midway between the patella and the iliac crest; the score is the number of times the right knee reaches the required height.

#### 2.5.4. Chair Sit-and-Reach Test

*Purpose*. Its purpose is to assess lower-body flexibility, which is important for good posture, normal gait patterns, and various mobility tasks, such as getting in and out of a bathtub or car.

*Description*. The patient is seated in a chair with legs extended. He/she was instructed to keep the back straight and reach the toes with both hands. The number of inches (centimeters) between the extended fingers and the tip of the toe was measured.

#### 2.5.5. Back Scratch Test

*Purpose*. Its purpose is to assess upper-body (shoulder) flexibility, which is important in tasks such as combing one's hair, putting on overhead garments, and reaching for a seat belt.

*Description*. The patient put one hand over the same shoulder with the palm touching the back and reached down the back. He/she placed the other hand up the back from the waist with the palm facing outwards. Pointing the middle fingers of each hand towards each other, patient tried to touch the fingers of each hand in the middle of the back. The number of inches (centimeters) between the extended middle fingers was measured. The test was always done with the right hand over the shoulder and the left behind the back.

#### 2.5.6. 8-Foot Up and Go Test

*Purpose*. Its purpose is to assess the agility and dynamic balance important in tasks that require quick maneuvering, such as getting off a bus in time, getting up to attend to something in the kitchen, going to the bathroom, or answering the phone.

*Description*. It is the number of seconds required to rise from a seated position, walk 8 feet (2.4 meters), turn, and resume the seated position.

### 2.6. Statistical Analysis

The assumption of normality was checked using the Kolmogorov-Smirnov test. The *α* level was set at *p* < 0.05. A *t*-test for repeated measures was used for statistical analysis, with a significance level set at *p* < 0.05. The software used for statistical analysis was Prism-GraphPad 5.3.

## 3. Results

**Figure 2:**
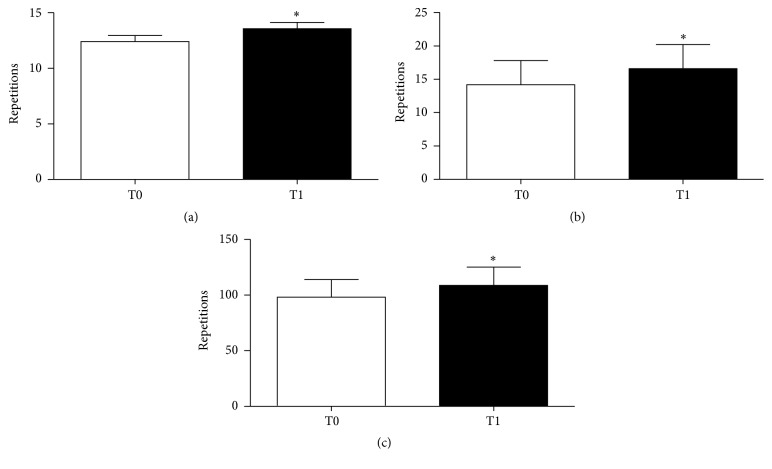
Comparison between the collected results at T0 and T1 during the Chair Stand (a), the Arm Curl (b), and the 2 min step (c). Values are mean ± SD. ^*∗*^ *p* < 0.05 versus T0.

**Figure 3:**
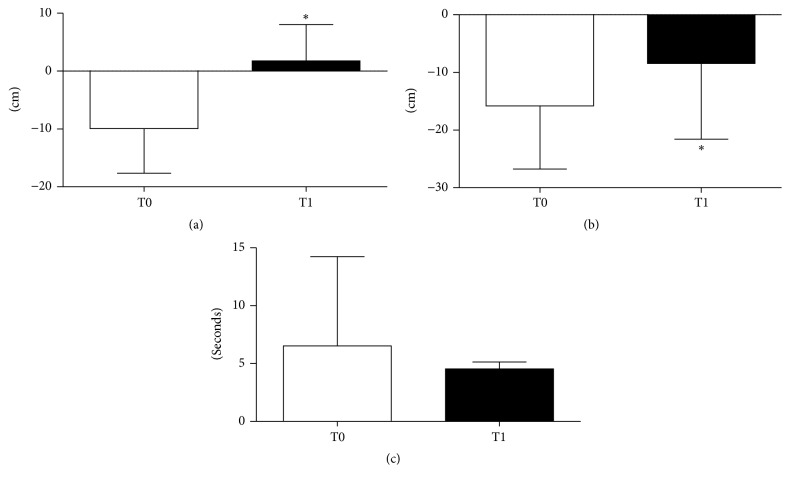
Comparison between the collected results at T0 and T1 during the Chair Sit-and-Reach (a), the Back Scratch (b), and the 8-foot up and go (c). Values are mean ± SD. ^*∗*^ *p* < 0.05 versus T0.

The VE group consisted of 8 women and 12 men (age 69.6 ± 3.9 years; weight 70.7 ± 12.1 kg; height 161.3 ± 6.9 cm). The control group consisted of 6 women and 14 men (age 71.2 ± 3.7 years; weight 76.1 ± 12.3 kg; height 167.5 ± 9.8 cm). Only 20 subjects of the VE group and 8 of the control group correctly completed the trials (see Figure 1 and* Limitation of the Study *paragraph). Adherence to protocol of the VE group was checked daily by our motor scientist by means of a daily record where he noted the week and participation number, the mean HR of the sessions, the type of exercises, and the number of repetitions per set carried out. During the training period, no adverse events such as dizziness, musculoskeletal pain, or cardiovascular issues were recorded. After 12 weeks, there were significant improvements in strength, flexibility, balance, and agility tested by SFT. T0-T1 differences are shown in Figures 2 and 3. Namely, 5 tests out of 6 showed significant improvement: Chair Stand (T0 12.4 ± 2.4; T1 13.5 ± 2.6, *p* < 0.01), Arm Curl (T0 14.2 ± 3.6; T1 16.6 ± 3.6, *p* < 0.01), 2 min step (T0 98.2 ± 15.7; T1 108.9 ± 16.2, *p* < 0.01), Chair Sit-and-Reach (T0 −9.9 ± 7.7 cm; T1 1.7 ± 6.3 cm, *p* < 0.01), and Back Scratch (T0 −15.8 ± 10.9 cm; T1 −8.4 ± 13.1 cm, *p* < 0.01). Conversely, the 8-foot up and go test (T0 6.5 ± 7.6 sec; T1 4.5 ± 0.6 sec, *p* > 0.05) showed no significant statistical difference due to a high SD in T0 assessment.

## 4. Discussion

The novelty of the present study is that of demonstrating the possibility of applying a specific vigorous physical exercise program [17] on healthy elderly adults over 65 years and evaluating its effects on functional capacity using the classical SFT [3]. To administer the high exercise intensity, we used a HR control under continuous accurate visual monitoring by a sport scientist. As expected, after only 12 weeks of training, we found significant enhancements of almost all skills tested. Our results clearly show that our VE program is relevant and has a positive impact on people over 65 in helping them to maintain a high quality of life. The difference from most of the literature [18–22] regards the exercise protocol intensity, which is usually milder than ours. Also, in the aforementioned studies there was a poor attention about the consequences of the exercise program on general quality of life of subjects. They mainly focused on the attenuated risks of falling. On the contrary, the SFTs applied in our study clearly show that our VE program may ameliorate several motor abilities and in turn the general quality of life in healthy elderly adults over 65 years of age. However, two other studies showed that elderly people need to exercise close to their limit of maximum capacity [23, 24] to improve their physical fitness but, unlike the present research, they were conducted on patients who were in deconditioning status linked to their chronic illnesses.

Brovold et al. [7] supposed the importance of an exercise is based on a high-intensity and continuous monitoring model because in their research a nonmonitored home-based group did not improve their physical fitness as much as the monitored group that accomplished a high-intensity aerobic exercise adjusted by means of the Borg Scale and a musical pace [25]. However, Brovold et al. [7], despite an exercise protocol with a high-intensity aerobic interval (HIA), found a small effect on SFT. This may be due to the fact that the exercise protocol used by Brovold et al. [7] did not interact favorably with the skills tested by SFT. Thus, a positive relationship among vigorous physical exercise [17] or HIA exercise [7] and the functional abilities tested by the SFT is not fully evident. On the contrary, the vigorous exercise protocol used here enhanced 5 out of 6 of the SFT and seems to be more focused than the aforementioned one. The small effect of vigorous physical exercise through the 8-foot up and go test is not fully clear and may depend on several factors: (i) a large standard deviation at T0 due to the presence of two subjects who showed a very low functional capacity; (ii) inadequacy of the exercises to improve this ability; and/or (iii) inadequate sensitivity of an 8-foot up and go test. In a recent study by Furtado et al. [15] conducted on a large number of elderly females, even though the SFT was used at baseline and after 8 months from an intervention program of multimodal exercise training (3 days per week), not all skills tested were found improved. However, according to a meta-analysis [11] that included 18 different exercise studies, even a small positive effect can be considered to be of great value in this group of individuals who are at risk of further functional decline. In conclusion, the present study shows that vigorous physical exercise in healthy elderly people provides significant improvements in the majority of the different skills assessed by the SFT.

*Limitation of the Study*. One potential limit of the present study undoubtedly regards the limited number of subjects involved in the study and the operating loss of the control group. Unfortunately, too many participants of the latter did not satisfy the requirements during the study, thus impeding a comparative statistical approach. Further studies are therefore needed to confirm our conclusions, in particular with a larger sample and control group.

## 5. Conclusions

The positive trend shown here is an encouraging result in this population in relation to the possibility of increasing their ability in performing daily activities, reducing the occurrence of falls and potential femoral fractures. Further research is needed to understand how to design a vigorous exercise protocol, which may focus not only on aerobics but also on the different skills assessed by the SFT and which may include specific training sessions to enhance those particular skills, such as 8-foot up and go test. To maximize the functional/physical capacities of those over 65, a close link between high-intensity exercise and functional exercises is required. A mixed circuit training program including both kinds of the aforementioned exercises and measurable by SFT should be followed.
